# Surface Electromyography Applied to Gait Analysis: How to Improve Its Impact in Clinics?

**DOI:** 10.3389/fneur.2020.00994

**Published:** 2020-09-04

**Authors:** Valentina Agostini, Marco Ghislieri, Samanta Rosati, Gabriella Balestra, Marco Knaflitz

**Affiliations:** ^1^PoliToBIOMedLab, Politecnico di Torino, Turin, Italy; ^2^Department of Electronics and Telecommunications, Politecnico di Torino, Turin, Italy

**Keywords:** electromyography, EMG, locomotion, machine learning, clinical practice, rehabilitation, physical therapy, outcome measurements

## Abstract

Surface electromyography (sEMG) is the main non-invasive tool used to record the electrical activity of muscles during dynamic tasks. In clinical gait analysis, a number of techniques have been developed to obtain and interpret the muscle activation patterns of patients showing altered locomotion. However, the body of knowledge described in these studies is very seldom translated into routine clinical practice. The aim of this work is to analyze critically the key factors limiting the extensive use of these powerful techniques among clinicians. A thorough understanding of these limiting factors will provide an important opportunity to overcome limitations through specific actions, and advance toward an evidence-based approach to rehabilitation based on objective findings and measurements.

## Introduction

Walking is one of the most essential activities of daily living (ADL) (1). The study of muscle activity during locomotion is of the uttermost importance in clinics, in the management of patients suffering from a wide variety of different neurological (2), orthopedic (3, 4), and peripheral vascular diseases altering gait patterns (5). Examples of neurological patients that might benefit from a thorough examination of the dynamic muscle activity are those affected by Parkinson disease (PD) (6, 7), post-stroke (8), multiple sclerosis (MS) (9), and hemiplegic children after cerebral palsy (10–13). Examples of orthopedic patients that might benefit from having the same examination are patients after anterior cruciate ligament (ACL) surgery (14), total knee arthroplasty (TKA) (3), knee megaprosthesis after tumor bone resection (15), total hip arthroplasty (THA) (16), and patients chronically affected by low back pain (17). Peripheral neuropathy (PN) and peripheral artery disease (PAD) are two distinct but related conditions that affect diabetic patients, altering their gait patterns up to the point of causing them foot ulcers often difficult to treat (“diabetic foot”) (5, 18). In the more severe cases, this can even lead to leg amputation.

Instrumented gait analysis provides comprehensive data on normal and pathological gait, which are useful in clinical practice producing objective information about time-distance variables (spatio-temporal data), joint motions (kinematics), and joint moments and powers (kinetics) (19). In the last decade, simplified, “user-friendly” techniques for gait analysis such as those based on accelerometric sensors are demonstrating their usefulness in the clinical setting and have had a significant impact in the literature (20–22). In addition, dynamic electromyography (EMG) allows for obtaining the timing and action of muscles, contributing to outline the patient's walking pattern and an empirical basis for identifying the functional cause of a gait abnormality (19).

Indeed, the knowledge about the dynamic contractile activity of the muscles during pathological gait may provide unique information to help clinicians in the following activities:

- to support diagnosis (2, 23)
- to design complex surgical interventions [e.g., multilevel surgery of hemiplegic children (24, 25)]
- to design personalized rehabilitation protocols and objectively prove their effectiveness (e.g., outcome evaluation of a proprioceptive training in MS patients), including new rehabilitation trends exploiting exoskeletons, e.g., in acute stroke patients (26), neurorehabilitation with Functional Electrical Stimulation (FES) (27), and any other system providing biofeedback based on myoelectric control (28–31)
- to support clinical decision (e.g., appropriate candidate selection for botulin toxin injection and choice of the target muscles (32), evidence-based choice of the type of joint prosthesis to implant (15)
- for therapy evaluation (e.g., to assess the effects of levodopa, or Deep Brain Stimulation on the muscle activation and muscle synergies of PD patients) (33– 35)
- for the production of quantitative reports to optimize patient's follow-up or to conduct longitudinal studies (16)
- to evaluate muscle fatigue (e.g., in ergonomics and sports) (36–39)
- to support forensic medicine with objective outcomes (e.g., to help medical insurance companies estimating a patient's risk, establishing adequate insurance compensations, unmasking simulators and avoiding frauds) (40, 41).

Despite the wide variety of possible clinical applications described above and their unquestionable relevance, clinicians underutilize instrumented gait analysis (GA) (42), especially associated to surface myoelectric signal detection (43–45). Surface electromyography (sEMG) is a well-established technique to investigate muscle activity non-invasively (46–48). In spite of that, clinicians rarely exploit the benefits of performing a “richer” and more complete gait analysis that includes, in addition to the analysis of the traditional spatio-temporal gait parameters and joint kinematics, the study of the muscle activation patterns during gait. Although underappreciated, the electrical activity of the muscles can be observed and recorded easily and non-invasively during locomotion (2, 49).

In the following, we will indicate with the acronym sEMG-GA gait analysis when it includes the recording of sEMG signals for sensing muscle activity during locomotion. SEMG-GA requires the acquisition of sEMG signals from the main lower limb muscles and, in some cases, from the trunk (50). The arm swing activity is more rarely reported, although it may be of clinical interest [e.g., PD patients may show a reduced arm swing activity during gait, in one or both sides (51)].

**Figure 1:**
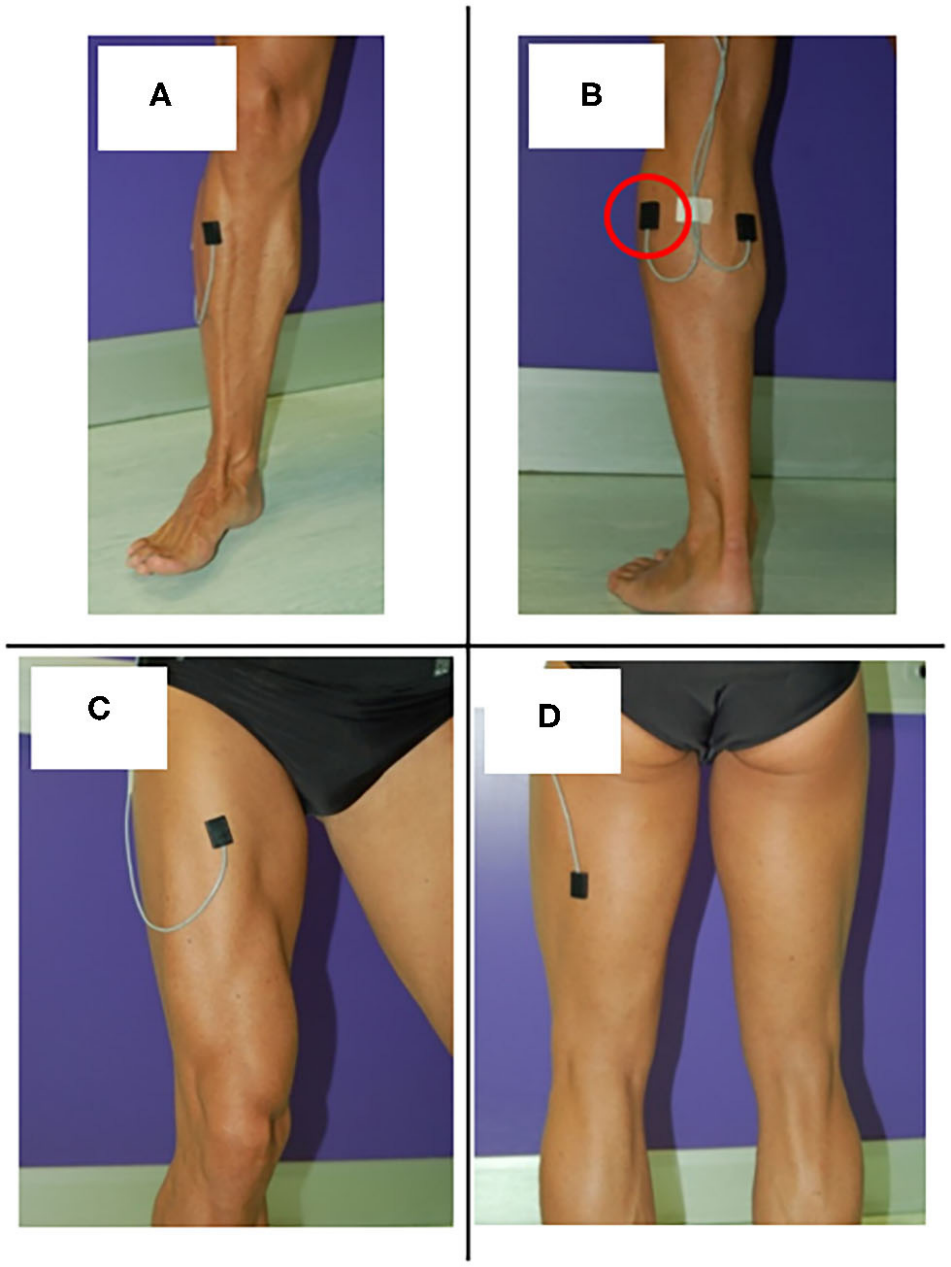
Surface EMG probes positioned over **(A)** Tibials Anterior (TA), **(B)** Lateral Gastrocnemius (LGS), **(C)** Rectus Femoris (RF), and **(D)** Lateral hamstrings (LH).

**Figure 2:**
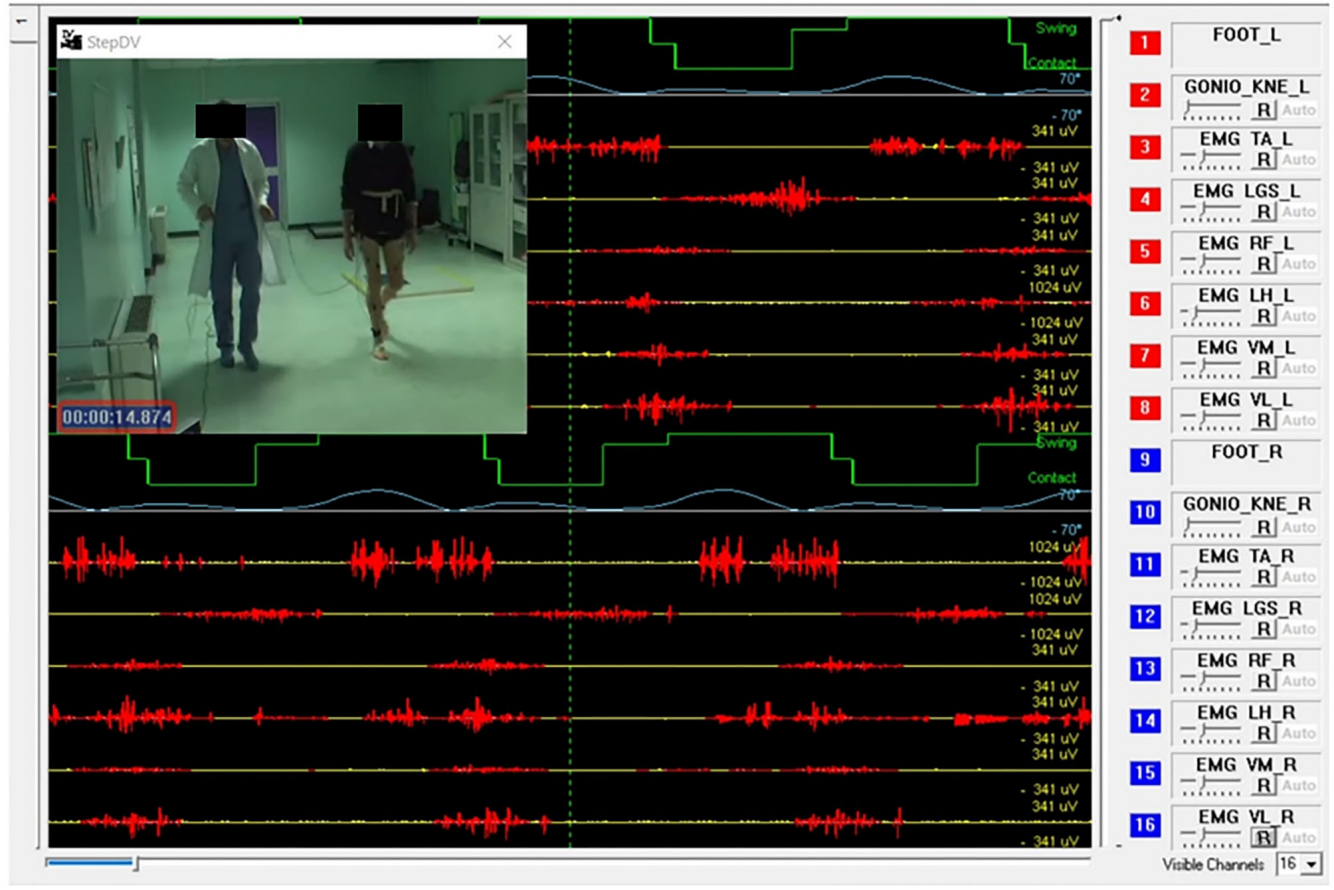
Example of signals acquired during a gait analysis session (multichannel recording system: STEP32, Medical Technology, Italy). Sixteen channels are synchronized with a video recording of gait: 8 for the left side (channels from 1 to 8) and 8 for the right side (channels from 9 to 16). For each lower limb the screenshot shows: 1 foot-switch-signal (green), 1 knee joint-angle kinematic signal in the sagittal plane (light blue), 5 sEMG signals over Tibialis Anterior (TA), Lateral Gastrocnemius (LGS), Rectus Femoris (RF), Lateral Hamstrings (LH), and Vastus Lateralis (VL). For each muscle, the activation patterns are automatically recognized by the system, and displayed in red, while the background noise is yellow-colored.

In a standard sEMG-GA session (52–54), sEMG probes are placed, at least, over Tibialis Anterior (TA), Lateral Gastrocnemius (LGS), Rectus Femoris (RF), and Lateral Hamstrings (LH), bilaterally, as reported by Figure 1. This allows for analyzing at least a pair of agonist-antagonist muscles acting at each joint of both lower limbs (ankle: TA/LGS; knee: LH-LGS/RF; hip: RF/LH). Indeed, since both LGS and RF are bi-articular muscles, this configuration makes it possible obtaining relevant biomechanical information using a minimum set of sEMG probes. SEMG signals can be acquired synchronously with foot-switch signals, joint kinematic signals, and a video recording (55). Figure 2 provides an example of signals acquired during a typical recording session performed using the multichannel recording system STEP32 (Medical Technology, Italy) (53). In this example, 16 channels with gait signals are synchronized with a video recording: 8 for the left side (channels from 1 to 8) and 8 for the right side (channels from 9 to 16). For each lower limb, the user-interface shows, in the same screenshot: the foot-switch signal, the knee joint-angle kinematic signal in the sagittal plane, and the sEMG signals over TA, LGS, RF, LH, and Vastus Lateralis (VL) muscles, respectively. For each muscle, the activation patterns are automatically recognized by the system, and re-visualized in red (distinguished from background noise, which remains yellow-colored).

A sEMG-GA test requires, overall, from 15 to 30 min (including sensor positioning). It is well-tolerated by children, adults, and the elderly, and by patients affected by a wide variety of pathologies altering locomotion patterns (2, 16, 18, 52, 56–63). The only requirement is the ability to walk independently for a few minutes. The exam can be carried-out also if the patient needs some walking aid or support (64), but, in this case, results must be carefully interpreted considering the specific situation. SEMG-GA is able to evidence even subtle gait abnormalities or gait pattern changes that are not perceivable at the naked eye by the clinician, in addition to “macroscopic” alteration or modifications of gait patterns. A possible application of sEMG-GA is the early evaluation of the effectiveness of a rehabilitation program (65, 66). Using sEMG-GA, clinicians will be able to obtain measurable outcomes after a few weeks of rehabilitation, even if only sub-clinical changes are present. In this manner, both the clinician and the patient will have a documented evidence that the rehabilitation program is working as expected or that it needs to be re-designed, if it did not lead to any measurable improvement. Therefore, performing sEMG-GA test during the patient's follow-up may also improve patient motivation and compliance to the rehabilitation program.

Yet, although there is a relevant number of studies supporting the use of sEMG in clinical gait analysis (2, 3, 16–18, 54, 60, 67), they seldom translate into routine clinical practice. The aim of this contribution is to critically analyze the key factors limiting the widespread use, among clinicians, of powerful techniques of clinical gait analysis based on sEMG signals. Possible solutions will also be outlined and discussed.

## Analysis of the Main Factors Limiting the Use Of sEMG-GA in the Clinical Practice

In this section, we discuss the following key factors limiting the widespread use of the sEMG signals in clinical gait analysis:

- lack of normative (reference) data of sEMG patterns [section Lack of Normative (Reference) Data Regarding sEMG Patterns]
- low intra-operator repeatability and inter-operator reproducibility in the collection of high-quality sEMG signals (section Low Intra-operator Repeatability and Inter-operator Reproducibility in the Collection of High-Quality sEMG Signals)
- inappropriate use of treadmill (instead of overground natural walking) (section Inappropriate Use of Treadmill (Instead of Overground Natural Walking)]
- difficulties in the sEMG-GA interpretability due to the large intra-subject variability of myoelectric patterns (section Difficulties in the sEMG-GA Interpretability Due to the Large Intra-subject Variability of Myoelectric Patterns)
- lack of reliable/compact/unique clinical scores obtainable from sEMG-GA (section Lack of Simple/Compact/Unique Clinical Scores Obtainable From sEMG-GA)

and discuss their characteristics and criticalities. The pinpointing of these limiting factors are the result of a 15-year experience of cooperative work and tight collaboration with clinicians of different specialties (neurologists, orthopedic surgeons, neurosurgeons, physiatrists, rehabilitation therapists, diabetologists…), in different gait analysis laboratories (hosted by hospitals, medical ambulatories, clinics, rehabilitation centers, gyms), with the aim of solving the research and clinical questions they had through sEMG-GA systems.

For each of these limiting factors, we also present, when available, possible solutions to overcome the described criticalities. When solutions are not currently available, we suggest future developments that might help bridging the gap between academic knowledge and clinical practice.

### Lack of Normative (Reference) Data Regarding sEMG Patterns

When a physician has available a sEMG-GA report, the first question that comes to his/her mind is: “How do I interpret this exam?” To provide a satisfying answer to this fundamental question, normative (reference) data on healthy populations are necessary. These normative data should be available, for each age class (children, adolescents, adults, elderly), differentiated by gender and body mass index (BMI). However, there is a lack of open databases of “physiological” sEMG activations patterns. One study analyzed 100 typically developing children aged 6–11 (52). Another study analyzed 40 healthy subjects, 20 aged 6–17 and 20 aged 22–72 (59). Most frequently, in the literature, only small datasets of 15–20 healthy subjects can be found, typically involving individuals recruited to build a control population (e.g., patient caregivers) related to a specific pathological target population (PD patients, diabetic patients…), and selected for a specific study aim. Only a few studies focus on making available large datasets (larger than 100 subjects) of physiological muscle activation patterns during locomotion. Furthermore, different sEMG acquisition systems and acquisition protocols are used, and there are different ways of processing and reporting data. Therefore, a standard is unavailable at the moment.

The authors suggest that the sEMG-GA systems to be used in clinics should be designed to automatically support clinicians with reliable reference data. Just as reference ranges (and eventually asterisks) appear on a blood test report, reference ranges should appear on a sEMG-GA report. Consequently, it is strongly advisable to integrate sEMG reference datasets into newly designed systems for clinical gait analysis. It would be ideal to produce these embedded reference datasets following the recommendation guidelines established through worldwide accepted standards, specifically developed for clinical gait analysis (67).

### Low Intra-operator Repeatability and Inter-operator Reproducibility in the Collection of High-Quality sEMG Signals

In a gait analysis laboratory, different professional figures may perform the acquisitions, such as biomedical engineers, gait analysis experts, physiatrists, physical therapists, and students. They have different expertise and some of them may lack experience in sEMG probe positioning, in recognizing the presence of detrimental artifacts in the signals, or in being aware of signal saturation or very low signal-to-noise ratio (SNR). This can lead to low inter-operator reproducibility. However, in clinical gait analysis, it is fundamental to guarantee that the different operators alternating in the various shifts do not affect the outcome measures derived from sEMG-GA. It follows that user-independent systems are required. Furthermore, it is also fundamental that the same operator is able to provide repeatable outcome measures, at different time-points, for a specific patient (e.g., to evaluate possible improvements after a therapeutic intervention).

A key factor to promote intra-operator repeatability and inter-operator reproducibility is the automatic assessment of the quality of the sEMG signals acquired, performed during the acquisition itself. However, there is a lack of systems designed to provide this essential feature (68). We suggest designing innovative systems that provide real-time information on the quality of each sEMG channel being acquired. These devices should help the training of less expert operators, independently from their background. As an example, to display the sEMG quality in real-time, a very intuitive semaphore's color coding might be used:

- GREEN: ok, good signal quality;
- YELLOW: sufficient, signal quality should be improved if possible;
- RED: completely inadequate, please stop the acquisition and check the electrodes.

### Inappropriate Use of Treadmill (Instead of Overground Natural Walking)

Frequently, sEMG signals are collected while the patient walks on the treadmill (12, 69, 70). This is often chosen merely for “tradition” (71), because it is “easier” for the experimenter (although not for the patient). Indeed, using treadmill allows confining the subject's cyclic motion to a small space-volume. This simplifies the acquisition protocol if there is the need to use a synchronized stereophotogrammetric system to detect gait events and jointly analyze 3D kinematics. Indeed, optical motion-capture systems were considered as the gold standard in the past, but they require to be calibrated over small sample volumes, in a confined lab space (72). Another “historical” reason why researchers frequently use the treadmill to study human gait is the possibility to obtain more controlled conditions, e.g., the possibility to set the velocity or the inclination to a pre-defined value.

However, the use of treadmill to study gait in pathological subjects may be rather inappropriate. Indeed, patients affected by neurological or musculoskeletal pathologies are not always able to walk on a treadmill, or it could be unsafe testing them on a treadmill, since additional balance skills are required to walk on a treadmill with respect to walk overground, naturally, at self-selected speed. Furthermore, when on the treadmill, the use of harnesses, or the fact that the patient, to maintain balance, leans on the treadmill horizontal bars or grasps vertical bars alters patient's perception, proprioception and muscle activation patterns. In addition, also if dynamic balance can be properly maintained without any external help, the muscle activations patterns during natural and treadmill gait are not the same (73). It was also demonstrated that the coordination between upper- and lower-limb movements is different during overground and treadmill walking (74). Hence, the possibility to perform sEMG analysis during “physiological” overground walking, instead of using a treadmill, can be important from a clinical point of view. This is something that should be carefully considered in the design of systems for clinical gait analysis.

Already 15 years ago, our research team designed a multichannel recording system for clinical gait analysis that integrated this design concept. The system was technologically transferred to an Italian company to reach the market (STEP32, Medical Technology) and it is being sold mainly in Italy and Spain. Thanks to this device, the possibility to perform sEMG-GA in the clinical setting, during overground walking, was fully demonstrated by several works (11, 16, 52, 63, 75, 76).

In recent years, the market revolution around wearable sensors based on Inertial Measurements Units (IMUs) has taken hold and is trying to substitute traditional motion capture systems with new devices, allowing for out-of-the-lab and low-cost motion analysis (77–79). We expect that this will reduce the use of treadmill in favor of the overground study of locomotion. Therefore, it seems promising to integrate wireless sEMG probes with IMUs to probe the dynamic muscle activity during overground locomotion, while reconstructing gait events and 3D joint kinematics. We think that such integrated wearable systems might greatly increase the use of sEMG-GA analysis in hospitals, rehabilitations centers and assisted-living facilities.

### Difficulties in the sEMG-GA Interpretability Due to the Large Intra-subject Variability of Myoelectric Patterns

It is well-known that human locomotion is characterized by a high intra-subject variability (80). Each gait cycle is different from the other, when muscle activation patterns are analyzed. Even in individuals with physiological walking patterns, sEMG activations noticeably vary from stride to stride (81). The sEMG variability can further increase in pathological subjects (11). This is the main reason why previous literature in clinical gait analysis discouraged analyzing a few gait cycles, and, it rather suggested analyzing “long” natural walks, lasting at least 3–5 min (16, 60, 82). Indeed, analyzing prolonged overground walks, carried out at natural pace, has been a successful strategy to obtain repeatable and reliable outcome measures, both in normal and pathological gait. However, this requires the use of advanced techniques of sEMG processing to automatically analyze hundreds of strides. Furthermore, if appropriate post-processing algorithms are not applied, the results obtained are cumbersome and the interpretation of muscle activation patterns becomes difficult or even impossible.

In the following, we will analyze various issues related to the sEMG gait variability and how it can make it difficult to interpret sEMG-GA, if not properly handled. In particular, we will distinguish between *extrinsic* and *intrinsic* sources of sEMG variability.

#### Extrinsic Sources of sEMG Variability: The Walking Track and the Need to Time Gait Events

Among the problems to tackle for analyzing a natural walk lasting several minutes, there is the fact that the acquisition should be performed, at least in theory, along a straight walking track between 200 and 500 m of length. However, this is unfeasible in many practical situations, for both technical and logistic issues, and it would require outdoor pathways. A reasonable solution is to have available, indoor, a large room or a long corridor (of length 10–15 m), which is not difficult to obtain in a hospital setting. Therefore, the patient can walk continuously, without interruptions, back and forth along the corridor. When arrived at the end of the walking track, the patient simply turns, reverses his/her direction, and keeps on walking, for many rounds. At each round, the patient travels for 10–12 gait cycles along the straight path, at an approximately steady velocity. Walking uninterruptedly for several minutes allows the patient to walk naturally, as in everyday life. Indeed, after a few rounds, the patient feels at ease and walks at his/her natural pace. Then, the signal acquisition can start.

**Figure 3:**
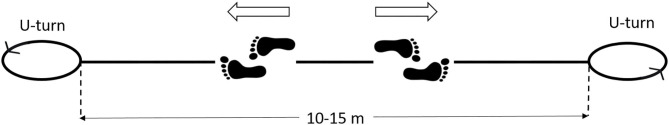
Scheme of the walking protocol for gait analysis. The patient walks back and forth, without interruptions, along a straight path of 10–15 m, for 3–5 min. The U-turns must be automatically removed from the analysis.

To process gait signals during overground walking, the first step is to segment gait cycles occurred during straight steady-state locomotion, separating them from the cycles relative to the direction changes, including decelerations before, and accelerations after the U-turns. Figure 3 shows this concept. In this way, gait parameters can be calculated in a repeatable manner, ruling out a first source of sEMG variability. However, it should be noticed that not only the U-turns, and their surroundings, must be discarded from the analysis, but also any other possible signal-epoch outliers, such as those corresponding to the abrupt distraction or sudden stop of the patient for any reason, or the unexpected change in his/her walking style that may happen along the walk. This issue can be properly handled if additional signals for timing gait events are collected, synchronous to the sEMG signals. These signals can be acquired through: (1) *indirect* measurements, by using stereophotogrammetric systems or wearable IMU sensors; (2) *direct* measurements, by using sensorized mats, foot-switches or foot-pressure insoles.

##### Indirect measurements to time gait events

As mentioned above, sterephotogrammetric systems have been historically considered the gold standard in gait analysis, both with and without an associated sEMG investigation. However, they never truly succeeded to help medical practitioners in clinical gait analysis, and most of the research-work done remained confined to academic studies. Indeed, stereophotogrammetric systems are expensive, they require a dedicated gait analysis laboratory and technical personnel, their sample volume is intrinsically limited to a few cube-meters, and they are complex to use, necessitating highly trained experts (typically biomedical engineers) to manage the system calibration and acquisition procedures. On the other hand, IMU systems are experiencing a “market boom” in many different applications, since they are lightweight, low-cost, and wearable, allowing for out-of-the-lab applications. Researchers, as well as medical-device producers, are actually trying to improve the performances of IMU systems on the reconstruction of joint angle measurements and 3D biomechanical models, and to mitigate drift errors observed during gait analysis (55, 79, 83, 84).

##### Direct measurements to time gait events

For what concerns direct measurements systems for timing gait events synchronous to sEMG signals, foot-switches already demonstrated their high potentialities in terms of accuracy, versatility, and ease of use in the past decades (85). Like IMUs, foot-switches are low-cost, lightweight, and allow for unconstrained acquisitions. At this time, they are the most valid alternative for timing gait events in clinical sEMG-GA. Timing gait events directly through foot-switches, the gait signals acquired from the whole walk of 3–5 min can be divided into strides, identifying the start and end of each gait cycle. Furthermore, within each gait cycle, the sequence of gait phases and their duration can be obtained. Then, sEMG signals corresponding to straight steady-state gait cycles, can be extracted and further analyzed, while disregarding outliers cycles. This can be performed by applying appropriate classification algorithms to recognize each gait cycle sequence (“cycle typology”) (85), and multivariate statistical filters based on gait phase duration (Hotelling *T*-square test) (86, 87). It should be noticed that this can be performed both in physiological and pathological gait, without the need for pre-defined stride templates, or complex algorithm customization targeting specific pathologies.

More specifically, placing 3 foot-switches under the heel, the first, and the fifth metatarsal heads (the main contact points of the foot with the ground in a normal subject) it is possible to obtain a 4-level basography, as shown by the green lines in Figure 1. This allows establishing the sequence of foot-floor contact gait phases and their duration. In normal gait, the standard sequence of gait phases of a stride is Heel contact (H), Flat foot contact (F), Push-off (P), Swing (S). Therefore, HFPS is the name assigned to the “normal” gait cycle. The average duration of gait phases in young adults (53), expressed as percentage of gait cycle (% GC), is:

- H = 6.6 ± 2% GC
- F = 26.4 ± 4% GC
- P = 22.6 ± 4% GC
- S = 44.4 ± 4% GC

**Figure 4:**
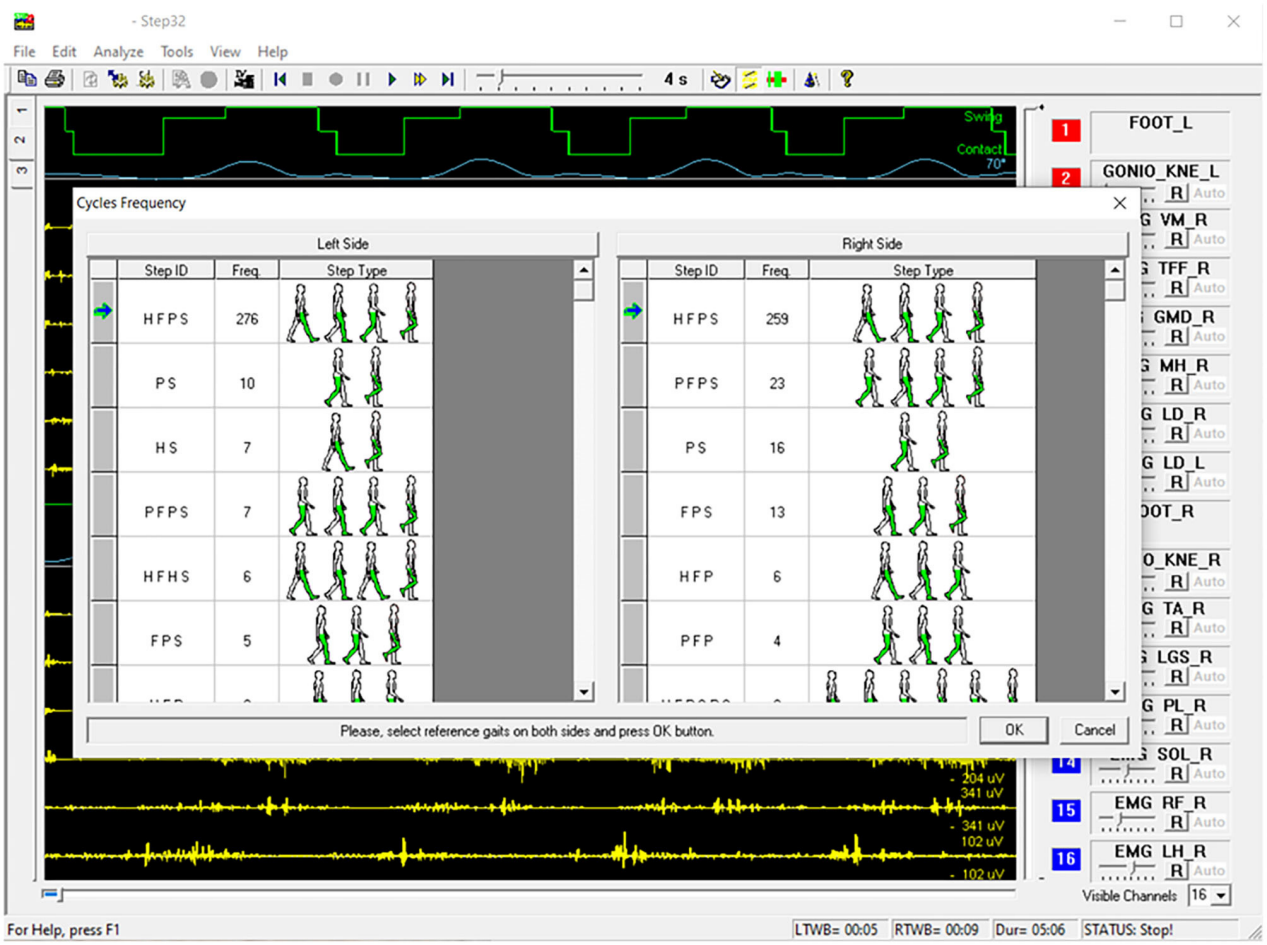
Example of STEP32 interface showing the automatic segmentation and classification of gait cycles for a walk lasting 5:06 min (Parkinson disease subject).

**Figure 5:**
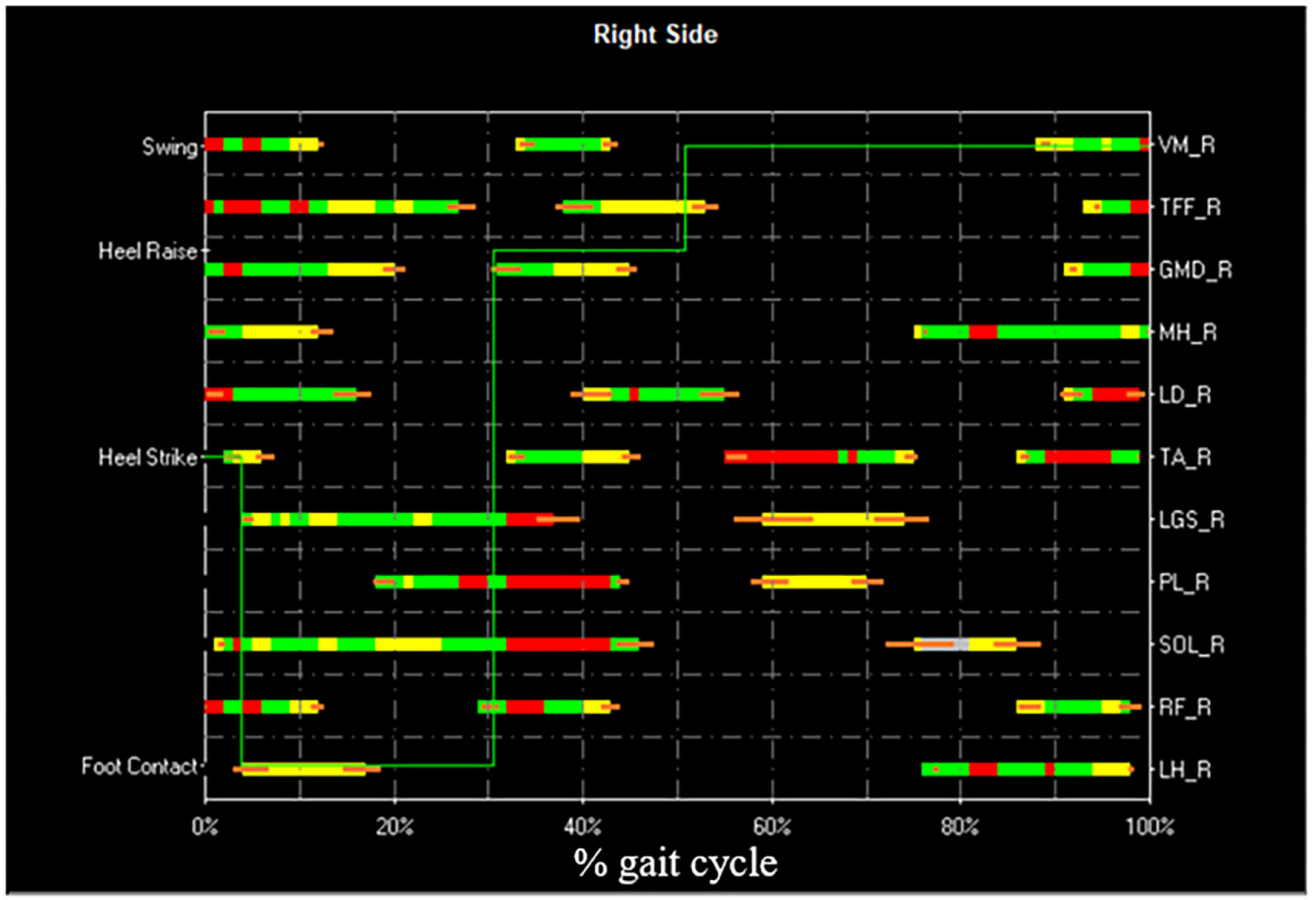
Example of STEP32 interface showing the results of the sEMG analysis. After the selection of the HFPS cycles reported in Figure 4, the most frequent activation patterns are shown for Vastus Medialis (VM), Tensor Fasciae Latae (TFF), Gluteus Medius (GMD), Medial Hamstring (MH), Longissimus Dorsii (LD), Tibialis Anterior (TA), Lateral Gastrocnemius (LGS), Peroneus Longus (PL), Soleus (SOL), Rectus Femoris (RF), Lateral Hamstring (LH) of the right side (most affected side of the PD subject). For each muscle, the orizontal bars represent the average activation intervals of the most frequent activation modality. The normalized amplitude is color-coded in three levels: high amplitude in red, medium amplitude in green, and small amplitude in yellow. Orange bars represent the standard error on the onset/offset detection of the activation intervals. The basographic signal is also shown superimposed (green line).

However, other gait cycle typologies are also observed, especially (but not exclusively) during U-turns. A markedly different sEMG activity is expected in these cases. Furthermore, the specific duration of gait phases, within a specific cycle typology, depends on the individual subject and gait speed, and it slightly changes from cycle to cycle. For each subject, and for each gait cycle type (e.g., HFPS), the mean, or the median value (more robust against outliers), of each gait phase duration can be calculated. In correspondence of the U-turns/outlier epochs, a relevant change in the sequence or in the duration of the gait phases appears in the basography and can be automatically detected. In pathological gait, the number of gait phases, their sequence, and duration can change with respect to normal gait, as well as the overall intra-subject variability. As an example, hemiplegic children after cerebral palsy, with a foot drop on the affected lower limb, typically strike the floor with the forefoot instead of the heel. They mainly show PFPS and/or PS sequence of gait phases instead of HFPS (82). Nevertheless, in the same manner as for healthy subjects, proper algorithms can handle gait cycle segmentation and classification, discarding outlier cycles, based on a statistical analysis (85). This approach is known as Statistical Gait Analysis (SGA) and it was developed and validated by our research group, specifically to deal with the challenges of clinical gait analysis mentioned above, to analyze hundreds of gait cycles in a user-independent way. The software of the STEP32 system integrates this “SGA philosophy”. Figure 4 shows the user interface where the gait cycles are classified and sorted by their frequency of occurrence (for a PD subject). Then, only sEMG signals corresponding to the gait cycles sharing the same foot-floor contact sequence are considered (HFPS was selected in this case). Figure 5 shows the results of the sEMG analysis.

We would like to stress that, if the subject contacts the floor differently in different gait cycles (e.g., with the forefoot instead of with the heel), it is evident that different sEMG patterns are produced. These differences are more pronounced in the distal part of the lower limb, e.g., for the ankle flexo-extensor muscles (TA and LGS). If this source of extrinsic sEMG variability is not properly handled, the results of the analysis cannot be accurate. Hence, a fundamental step before analyzing sEMG patterns is to group together only those patterns belonging the same typology of gait cycle.

While a gait analysis expert can select the subject's most representative gait cycles, choosing them one-by-one “manually,” this is unfeasible in clinical applications, requiring a reliable and repeatable analysis of hundreds of gait cycles, in a user-independent manner. Therefore, in summary, it is advisable that systems designed for clinical sEMG-GA incorporate algorithms to:

- remove U-turns and outlier epochs
- segment and classify gait cycles and their frequency of occurrence
- focus sEMG analysis on representative gait cycles of the same type.

#### Intrinsic Sources of sEMG Variability

**Figure 6:**
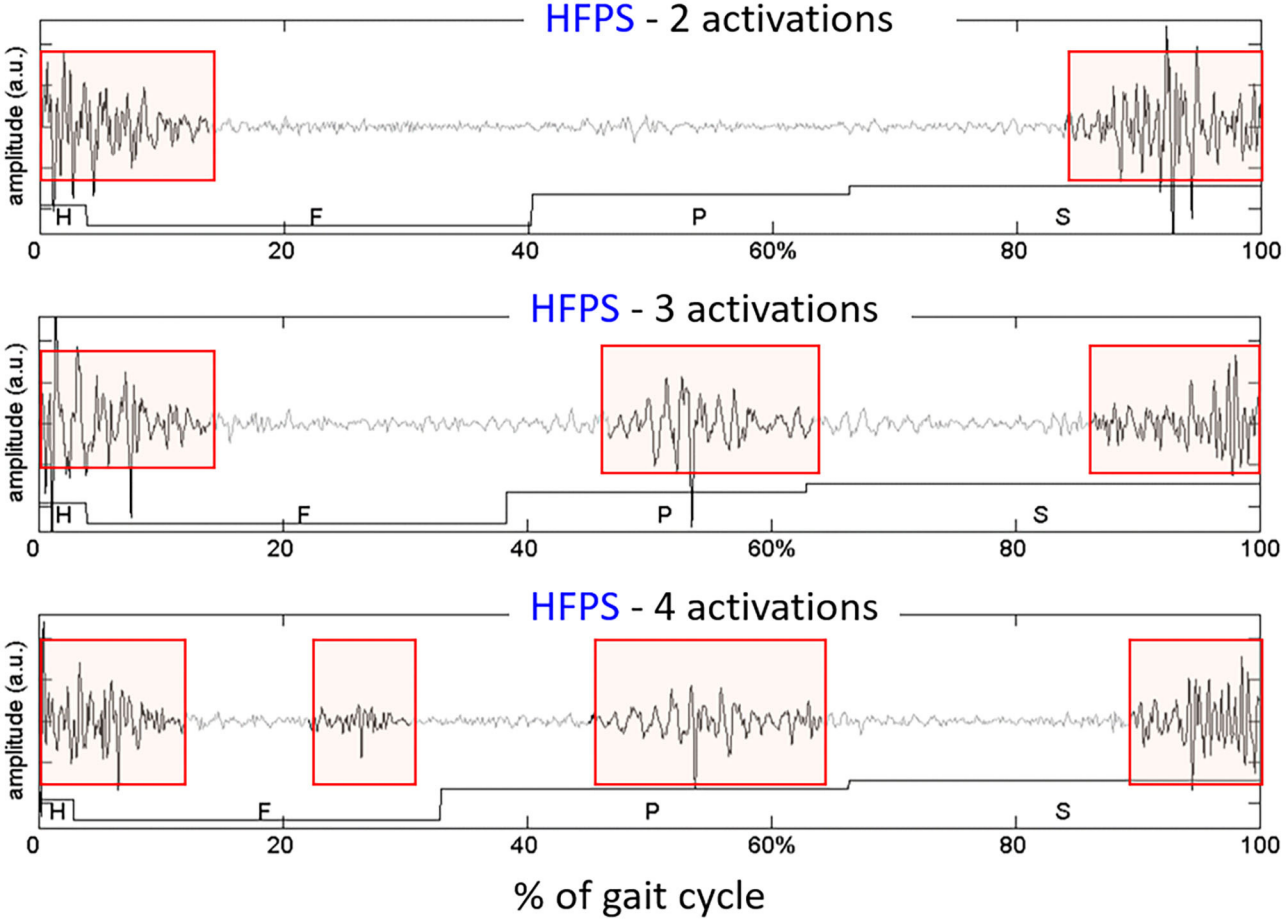
Example of different activation modalities of the Rectus Femoris, observed in sEMG signals collected from an healthy subject, on 3 gait cycles of the same type (HFPS) collected during the same walk. The colored boxes highlight the intervals in which the muscle activity is present.

Even if sEMG signals are processed separately for each class of representative gait cycles, there are other sources of intra-subject variability that must be accounted for. More specifically, literature reports that, even in normal HFPS gait cycles of healthy subjects, a specific subject's muscle does not show a single “preferred” pattern of activation. Instead, from 3 to 5 distinct sEMG patterns are usually observed, each characterized by a different number of activation intervals occurring within the gait cycle. These are called “activation modalities” (52). Figure 6 shows sEMG variability on a representative subject (young healthy individual). However, especially when analyzing pathological subjects, inspecting separately each modality of activation and its frequency of occurrence (88) may be rather cumbersome. Consequently, clinicians may lose interest, since results are difficult to interpret.

**Figure 7:**
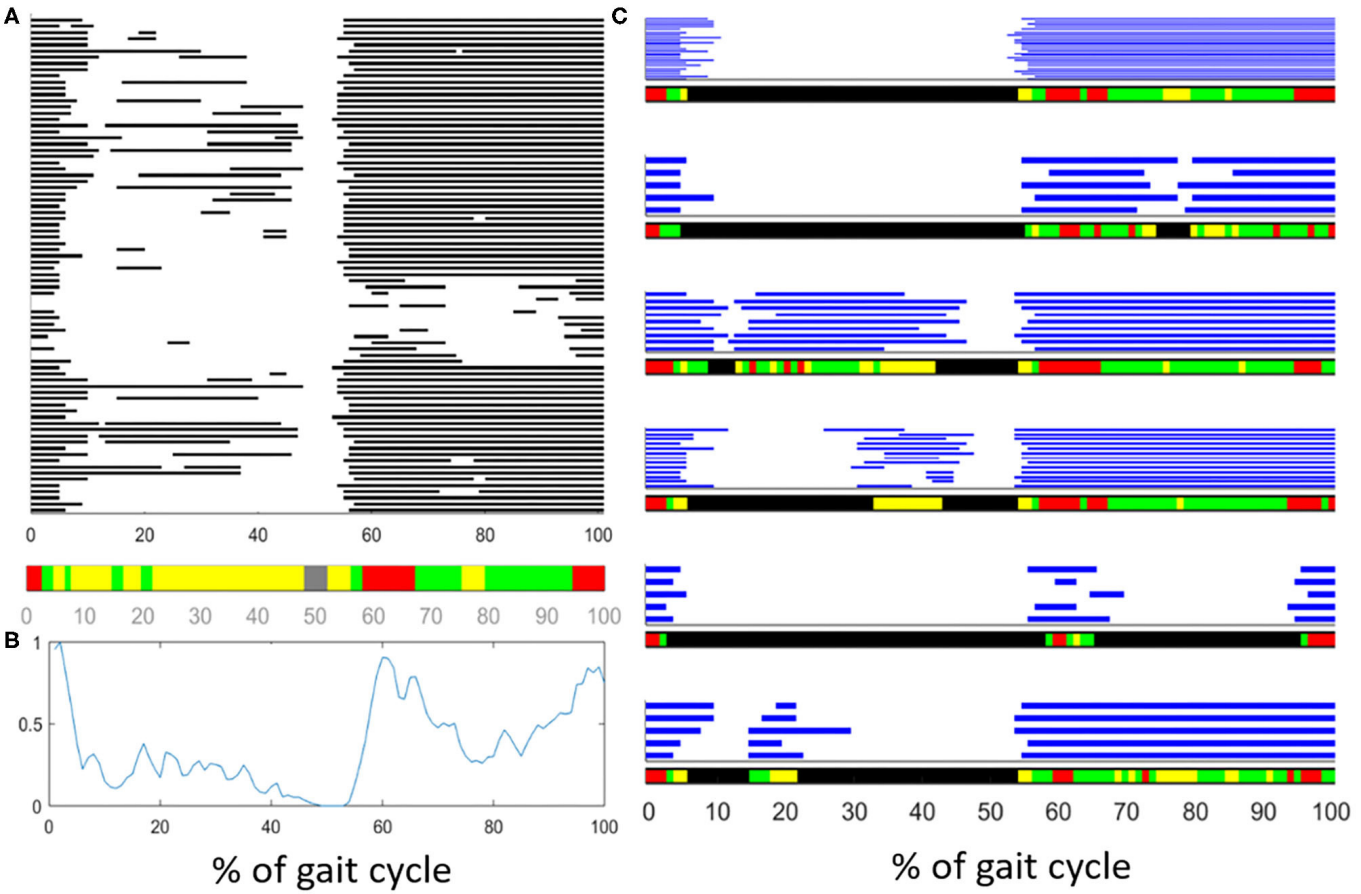
Description of the effects of intrasubject variability on the interpretation of muscle activation patterns during gait. **(A)** Example of variability in the activationd of the Tibialis Anterior (TA) muscle of an healthy subject. Gray bars represent the activation intervals in different gait cycle of the same walk. Observing the average activity, represented in the color bar below **(A)** or represented as a “linear envelope” in **(B)**, one would conclude that TA muscle is almost always active. **(C)** After grouping together the gait cycles sharing the same activation-timing patterns, the average activation intervals (within each group) really represent the muscular activation patterns. In the latest case, the biomechanical task of each activation interval becomes clear.

**Figure 8:**
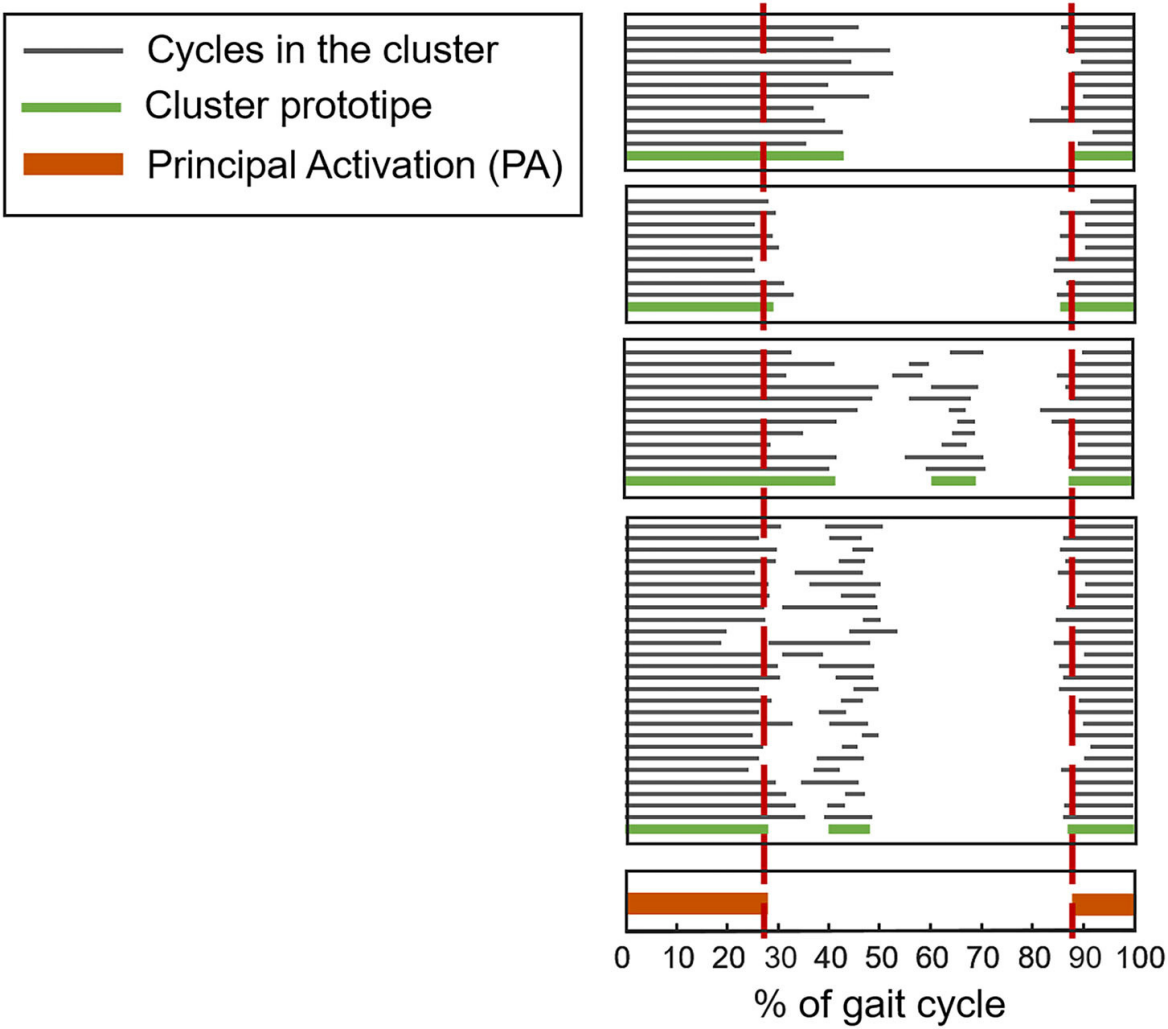
Example of application of CIMAP (Clustering algorithm for Identification of Muscle Activation Patterns) for the Rectus Femoris muscle. Gray bars represent the activation intervals in different gait cycles of the same walk. The Principal Activation (PA) is univoquely determined as the intersection of cluster's prototypes.

In recent years, a clustering algorithm was proposed and validated, both on healthy and pathological subjects, to manage intrinsic sources of within-subject sEMG variability in overground locomotion. This algorithm, named CIMAP (Clustering for Identification of Muscle Activation Patterns) allows grouping together sEMG patterns sharing similar timing patterns (58, 81, 89). Then, it is possible to define, in a unique manner, the sEMG principal activations (PA) of a subject during gait (63, 90–93). A single binary string, representing PA intervals, is associated to the overall dynamic activity of the muscles during a subject's locomotion (1: the muscle is active; 0: the muscle is non-active). Figure 7 shows the effects of intra-subject variability on the interpretation of sEMG activation patterns, graphically illustrating the importance of using clustering algorithms. Figure 8 schematically depicts the extraction of PA in a representative subject.

The use of this kind of algorithms would enormously simplify the interpretation of sEMG signals during locomotion. Indeed, a single (but representative) PA-string might be compared before and after a rehabilitation program, or a therapeutic intervention, helping clinicians in their work. Notice that this process is scalable, and can be repeated to obtain unique PA from a cohort population (instead of a single patient) to ease the interpretation of randomized clinical trials using sEMG measurements (91).

However, the described advanced sEMG processing tools, aimed at managing the intrinsic sources of sEMG variability, are currently not available to clinicians. Presently, there are no medical system integrating these important features. Future systems designed for clinical sEMG-GA should incorporate, cascaded to the algorithms mentioned in the previous section, CIMAP-like algorithms.

### Lack of Simple/Compact/Unique Clinical Scores Obtainable From sEMG-GA

After many years of tight collaborative work with clinicians, and fervent requests of help in the interpretation of sEMG signals during gait, we understood that an essential point is providing simple/compact/unique clinical scores for easily comparing the patient outcome at different time points (and with reference data).

The great majority of research papers typically presents results from dozens of different parameters, typically estimated from signals of each specific muscle under study. This makes the application to patient management difficult. A few attempts can be found in literature to summarize the information obtained from sEMG-GA test in a unique and representative value of the patient's locomotion performance. One successful recent example is the introduction of a sEMG-based “asymmetry index” (54). This index defines the patient's global asymmetry during gait, and it was validated on different orthopedic populations (total hip arthroplasty, total knee arthroplasty) and neurological populations (hemiplegic children, normal pressure hydrocephalus). Furthermore, reference values for different age populations (children, adults, elderly) were also provided.

However, none of the available systems for sEMG-GA integrates this index or similar ones.

## Discussion and Conclusions

One of the fathers of modern sEMG analysis, Prof. C.J. De Luca, already 30 years ago warned that the sEMG signal, if not properly analyzed, could become “a seductive muse.” In the last decade, there have been intense efforts to find reliable methods to process and correctly interpret muscle activation patterns in locomotion. Nevertheless, there is still an evident gap between literature findings and clinical practice. In this contribution, we critically analyzed the main key factors limiting the widespread use of sEMG signals in clinical gait analysis.

In synthesis:

- There is a lack of open databases related to reference populations (of healthy children, adults, and elderly) containing normative activation patterns as well as raw sEMG signals, collected during gait. Furthermore, there are no accepted standards on how to report muscle activation patterns.
- There is a lack of systems for clinical gait analysis that integrate quality information about the collected sEMG signals, in real-time, to improve intra-operator repeatability and inter-operator reproducibility.
- There is a lack of (wearable and wireless) systems for sEMG detection that integrate algorithms for the study of gait in natural conditions.
- There is a lack of systems that integrate algorithms aimed at managing the high intra-subject variability of sEMG patterns in human gait (both of extrinsic and intrinsic nature).
- There is a lack of systems that integrate simple scores or indexes, calculated from sEMG-GA data, to help clinical interpretation.

Therefore, the authors believe that it is fundamental to rethink this research field, organizing debates, consensus meetings, interdisciplinary projects and other initiatives to provide a critical view of the topic and, last but not least, redesign user-friendly systems for sEMG-GA, usable in clinics. In addition, it would be important to offer training on sEMG-GA techniques to clinicians and health practitioners, including open education and open data resources. If the proposed “positive actions” will be successful, good clinical practices will benefit from new evidence-based approach to rehabilitation.

## Data Availability Statement

The raw data supporting the conclusions of this article will be made available by the authors, without undue reservation.

## Ethics Statement

Ethical review and approval was not required for the study on human participants in accordance with the local legislation and institutional requirements. The patients/participants provided their written informed consent to participate in this study.

## Author Contributions

VA and MK designed the article structure and topics covered. VA drafted the article, with the contribution of MG for the production of some of the figures. MG, SR, GB, and MK critically revised the article. All authors contributed to the article and approved the submitted version.

## Conflict of Interest

The authors declare that the research was conducted in the absence of any commercial or financial relationships that could be construed as a potential conflict of interest.
